# Effectiveness of flower therapy in reducing anxiety in older adults

**DOI:** 10.15649/cuidarte.4887

**Published:** 2026-04-17

**Authors:** Karoline Galvão Pereira Paiva, Odézio Damasceno Brito, Thamires Sales Macêdo, Jamily Soares Damasceno da Silva, Ana Clécia Silva Monteiro, Lívia Moreira Barros, Natasha Marques Frota

**Affiliations:** 1 Universidade da Integração Internacional da Lusofonia Afro-Brasileira – UNILAB, Redenção, Ceará, Brazil. E-mail: karolinegalvaopaivajk@gmail.com Universidade da Integração Internacional da Lusofonia Afro-Brasileira – UNILAB Ceará Brazil karolinegalvaopaivajk@gmail.com; 2 Universidade da Integração Internacional da Lusofonia Afro-Brasileira – UNILAB, Redenção, Ceará, Brazil. E-mail: odeziod@gmail.com Universidade da Integração Internacional da Lusofonia Afro-Brasileira – UNILAB Ceará Brazil odeziod@gmail.com; 3 Universidade Federal do Ceará - UFC, Fortaleza, Ceará, Brazil. E-mail: thamiressales1998@outlook.com Universidade Federal do Ceará - UFC Ceará Brazil thamiressales1998@outlook.com; 4 Universidade da Integração Internacional da Lusofonia Afro-Brasileira – UNILAB, Redenção, Ceará, Brazil. E-mail: jamilysoares@hotmail.com Universidade da Integração Internacional da Lusofonia Afro-Brasileira – UNILAB Ceará Brazil jamilysoares@hotmail.com; 5 Universidade da Integração Internacional da Lusofonia Afro-Brasileira – UNILAB, Redenção, Ceará, Brazil. E-mail: clecia.monteiro_26@hotmail.com Universidade da Integração Internacional da Lusofonia Afro-Brasileira – UNILAB Ceará Brazil clecia.monteiro_26@hotmail.com; 6 Universidade da Integração Internacional da Lusofonia Afro-Brasileira – UNILAB, Redenção, Ceará, Brazil. E-mail: livia.moreirab@hotmail.com Universidade da Integração Internacional da Lusofonia Afro-Brasileira – UNILAB Ceará Brazil livia.moreirab@hotmail.com; 7 Universidade da Integração Internacional da Lusofonia Afro-Brasileira – UNILAB, Redenção, Ceará, Brazil. E-mail: natasha@unilab.edu.br Universidade da Integração Internacional da Lusofonia Afro-Brasileira – UNILAB Ceará Brazil natasha@unilab.edu.br

**Keywords:** Floral Therapy, Flower Essences, Mental Health, Anxiety, Elderly Health, Betty Neuman, Terapia Floral, Esencias Florales, Salud Mental, Ansiedad, Salud del Anciano, Betty Neuman, Terapia Floral, Essências Florais, Saúde Mental, Ansiedade, Saúde do Idoso, Betty Neuman

## Abstract

**Introduction:**

Anxiety can arise at different stages of life and is often exacerbated by feelings of distancing from family and friends. The use of integrative practices in older adults' care can complement conventional treatments by providing relief from pain, stress, and emotional difficulties.

**Objective:**

To analyze the effectiveness of flower therapy in treating anxiety among older adults.

**Materials and Methods:**

A quantitative, quasi-experimental, double-blind study was conducted between August 2022 and January 2023 with 59 older adults (control group = 28 and intervention group = 31). The intervention group used a combination of flower essences: Impatiens, Clematis, Star of Bethlehem, Cherry Plum, and Rock Rose, included in a formula and brandy-based tincture for anxiety. The control group continued with routine follow-up in primary health care. Data were collected using the State-Trait Anxiety Inventory in both groups at three time points.

**Results:**

The use of flower therapy significantly reduced anxiety in the intervention group compared to the control group, specifically in terms of impatience, fear, irritability, restlessness, agitation, and family-related worries, with p-values less than 0.001.

**Discussion:**

In the context of older adult care, integrative therapies have shown promise, promoting mental health improvements following flower therapy interventions.

**Conclusion:**

The use of complementary therapies, such as flower essences, reduces anxiety, fear, agitation, and irritability in older adults, offering an integrative alternative in health care.

## Resumen

**Introducción:**

La ansiedad puede manifestarse en diferentes etapas de la vida, muchas veces agravada por sentimientos de distanciamiento de la familia y los amigos. El uso de prácticas integrativas en el cuidado del adulto mayor puede complementar los tratamientos convencionales, proporcionando alivio del dolor, el estrés y las dificultades emocionales.

**Objetivo:**

Analizar la eficacia de la terapia floral en el tratamiento de ansiedad en adultos mayores.

**Materiales y métodos:**

Estudio cuasiexperimental, doble ciego, cuantitativo, realizado entre agosto de 2022 y enero de 2023, con 59 adultos mayores (grupo control = 28 y grupo de intervención = 31). El grupo de intervención utilizó una combinación de esencias florales *Impatiens, Clematis, Star of Bethlehem, Cherry Plum y Rock Rose*, presentes en una fórmula y solución de coñac para ansiedad. El grupo control continuó con el seguimiento rutinario en la atención primaria de salud. La recolección de datos se realizó mediante la escala State-Trait Anxiety Inventory en ambos los grupos, en tres momentos.

**Resultados:**

El uso de la terapia floral redujo significativamente la ansiedad en el grupo de intervención en comparación con el grupo control, en aspectos como impaciencia, miedo, irritabilidad, inquietud, agitación y preocupaciones familiares, con valores de p menores a 0,001.

**Discusión:**

En el contexto del cuidado al adulto mayor, las terapias integrativas se han mostrado prometedoras, promoviendo mejoras en la salud mental tras intervenciones con terapia floral.

**Conclusiones:**

El uso de terapias complementarias, como las esencias florales, reduce la ansiedad, el miedo, la agitación y la irritabilidad en adultos mayores, proporcionando una alternativa integrativa para los cuidados de salud.

## Resumo

**Introdução:**

A ansiedade pode surgir em diferentes fases da vida e é frequentemente exacerbada por sentimentos de desconexão com a família e os amigos. O uso de práticas integrativas no cuidado ao idoso pode complementar os tratamentos convencionais, proporcionando alívio da dor, do estresse e das dificuldades emocionais.

**Objetivo:**

Analisar a eficácia da terapia floral no tratamento da ansiedade em idosos.

**Materiais e métodos:**

Estudo quantitativo, quase experimental, duplo-cego, realizado de agosto de 2022 a janeiro de 2023, envolvendo 59 idosos (grupo controle = 28 e grupo intervenção = 31). O grupo intervenção utilizou uma combinação de essências florais: *Impatiens, Clematis, Star of Bethlehem, Cherry Plum e Rock Rose*, incluídas em uma fórmula com solução de conhaque para ansiedade. O grupo controle continuou com acompanhamento de rotina na atenção primária à saúde. A coleta de dados foi realizada por meio do Inventário de Ansiedade Traço-Estado em ambos os grupos em três momentos distintos.

**Resultados:**

O uso da terapia floral reduziu significativamente a ansiedade no grupo de intervenção em comparação ao grupo controle, especificamente em termos de impaciência, medo, irritabilidade, inquietação, agitação e preocupações relacionadas à família, com valores de p inferiores a 0,001.

**Discussão:**

No contexto do cuidado a idosos, as terapias integrativas têm se mostrado promissoras na promoção de melhorias na saúde mental após intervenções de terapia floral.

**Conclusões:**

O uso de terapias complementares, como essências florais, reduz a ansiedade, o medo, a agitação e a irritabilidade em idosos, oferecendo uma alternativa integrativa no cuidado à saúde.

## Introduction

Concerns regarding the longevity and vulnerability of older adults have been gradually increasing within the healthcare sector^1^. It is observed that anxiety is significantly present in this age group and is associated with familial and social distancing issues^2^. Studies indicate that the international prevalence of anxiety among older adults ranges from 1.2% to 15%, and it remains underdiagnosed and associated with functional decline. The main risk factors are insomnia, female sex, widowhood, social isolation, physical inactivity, chronic diseases, and low self-esteem. Older adults with anxiety experience functional deterioration and make more use of healthcare services^3^,^4^.

A cross-sectional study conducted in China reported exponential growth in anxiety symptoms among this population, corroborating increases in sedentary lifestyle, chronic diseases, insomnia, and gastroenteritis^4^.

Similarly, a Brazilian study identified increased anxiety and depression among older adults during the pandemic, with negative effects related to reduced physical activity, stress, increased screen exposure, and fear^5^. In this context, the study of integrative and complementary practices, which advocate holistic care in pursuit of better health conditions, has become consolidated^6^,^7^.

Integrative and complementary practices used with older adults are diverse and include homeopathy, phytotherapy, acupuncture, anthroposophy, flower essence therapy, medicinal plants, and thermalism^8^. Among these practices, flower therapy uses vibrational therapeutics of certain flowers, plants, and trees to balance negative thoughts and emotions, as well as personality disorders that are potential causes of diseases. By using flower essences, this therapy aims to balance the existential, spiritual, physical, and emotional dimensions of the individual^9^.

Thus, caring for people who suffer from anxiety through flower therapy may be an interesting therapeutic option, as illness is not restricted only to psychological issues^10^, especially among the older adult population.

Anxiety can manifest both as an initial protective response and as a more debilitating condition if not controlled^11^. Therefore, intervening in the anxiety of older adults through flower essences makes it interesting to observe how they affect the precursors of this condition, drawing on the metaparadigm concepts: nursing, person, health, and environment, within Betty Neuman's Systems Model to understand the stressors that lead to illness and propose timely interventions^12^.

There are precursors that appear as early signs of an anxiety disorder, such as cold hands, impatience, fear, tachycardia, sweating, and anger^13^. These physical and emotional signs can be explained as responses to internal and external stressors, according to Neuman's model^14^.

Results from other studies confirm the efficacy of flower therapy in reducing anxiety, improving sleep patterns, and decreasing stress levels^14^,^15^, encouraging further studies to evaluate these effects across various populations with an emphasis on promoting this topic.

Given the reported benefits of flower therapy and its influence on reducing anxiety, this study aimed to analyze the effectiveness of flower therapy in treating anxiety in older adults. The theoretical framework used was Neuman's model. To understand the origin of anxiety in light of Neuman's Systems Model, Hans Selye (1959) divides it into three phases that describe the assumptions about anxiety adopted in data analysis^12^.

## Materials and Methods

**Study design**

This prospective, double-blind, quasi-experimental study with a 1:1 allocation ratio evaluated the effectiveness of flower therapy on anxiety levels among older adults residing in the interior region of the State of Ceará, Brazil, from August 2022 to January 2023. The study followed the Consolidated Standards of Reporting Trials (CONSORT) guidelines.

**Sample and participants**

The study population comprised older adult patients receiving care in primary health care (PHC) services in Redenção, a city in the interior of Ceará, Brazil. Inclusion criteria were older adult patients of both sexes who were literate, active in their daily activities, and able to self-identify with at least three of the following self-reported traits or characteristics: anxiety, haste, impatience, irritability, impulsiveness, restlessness, difficulty relaxing, intolerance toward others' slower pace, nervousness, tension, fear, and loneliness.

Exclusion criteria included the use of other flower or complementary therapies, ongoing psychological treatment, alcoholism, and psychotropic drug use. Discontinuation criteria were non-adherence to therapy for five consecutive days, incorrect dosing, interruption of two sessions, or nonattendance at consultations.

For the sample size calculation to compare means between the control group (CG) and the intervention group (IG), 59 older adults were included: 28 in the CG and 31 in the IG. The sample was based on a previous study conducted by Santos et al.^16^, which used convenience sampling, with a statistical power of 80% and an alpha error of 5%. The calculation was based on an anxiety disorder prevalence of 20%, and, as a result, it was possible to determine that 58.5% of older adults exhibited some anxiety traits according to the criteria of Roy-Byrne et al.^17^ following the application of the State-Trait Anxiety Inventory (STAI).

**Randomization and blinding**

Allocation was performed in a 1:1 ratio to either the CG or the IG using simple randomization with the free online software Research Randomizer, and the results were stored in Microsoft Excel. This process was conducted by an external researcher to minimize the risk of bias. Participants, the researchers responsible for data collection at M1 and M2 time points, and the statistician remained blinded to group allocation to ensure blinding throughout the study.

**Figure 1:**
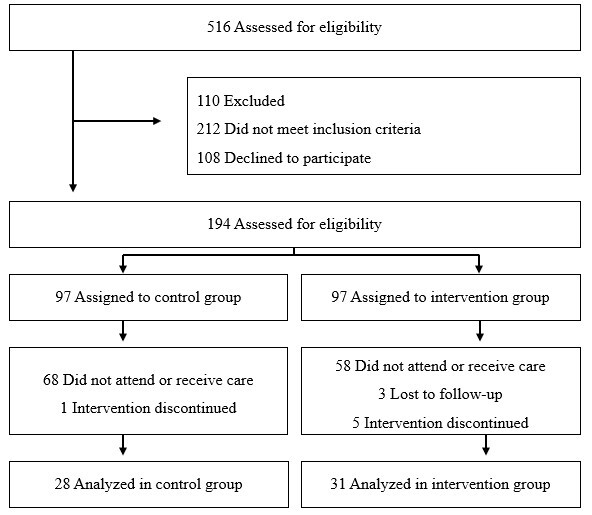
Recruitment flow of the clinical trial. Fortaleza, CE, Brazil, 2024

The flow of recruitment, exclusion, and allocation of participants is shown in Figure 1.

**Intervention**

The intervention in this study involved the administration of a flower essence tincture designed to mitigate anxiety symptoms in older adults. This tincture is a potent agent for emerging crises and one of the most studied and compared in terms of efficacy and effectiveness^17^. The flower essence formula was prepared and dispensed by a specialized homeopathic and flower essence compounding pharmacy that adhered to all regulatory and ethical guidelines, ensuring confidentiality, blinding, and production rigor.

Data were collected through coordination with PHC teams and the registration of older adults within the unit. Older adults were invited to participate in the study through invitation cards distributed by Community Health Agents (CHAs), announcements at the health unit, and community outreach dialogues. On scheduled data collection days, older adults who were present were randomized and allocated to either the IG or CG. Subsequently, each participant was taken to the nursing station, received instructions, and provided informed consent.

The intervention group (IG) received a 30-ml bottle containing a standardized combination of Impatiens, Clematis, Star of Bethlehem, Cherry Plum, and Rock Rose, in a diluted solution of 30% brandy, following standard flower essence therapy protocols.

Participants in the IG were instructed to take four drops per dose, administered sublingually at four key moments throughout the day: upon waking, before lunch, before dinner, and before bedtime. To ensure adherence to the intervention, participants were provided with a tracking card on which they recorded each administration. Participants were also instructed to avoid consuming the flower essence formula near mealtimes or immediately before or after brushing their teeth, as bold flavors could interfere with the tincture’s absorption.

The control group (CG) followed the same administration procedure but received a placebo tincture identical in appearance and taste, containing only diluted brandy without flower essences.

Participants were assessed at three different time points. At baseline (M0), before starting the intervention, a clinical-epidemiological questionnaire and the STAI were administered to measure initial anxiety levels. After 15 days (M1), a follow-up assessment was conducted to monitor adherence, adverse reactions, and any perceived effects. Finally, at 30 days (M2), participants were reassessed using the STAI state anxiety subscale to determine changes in state anxiety levels.

Following this schedule, instruments were applied in stages. The first instrument was the clinical-epidemiological questionnaire and vital signs measurement, and the second was the STAI, translated and adapted for Brazil^18^, which includes two subscales (trait anxiety and state anxiety) administered to both the CG and IG.

Throughout the intervention, any adverse events or significant changes in participants’ health conditions were monitored and reported. Participants who experienced any discomfort were advised to suspend use for 24 hours and seek further clinical evaluation if necessary. At the end of the study, both groups were informed of their original allocation. The flower essence formula was made available to the IG for continued use, and CG participants were offered the flower therapy, if they wished to try it.

**Data analysis**

The data were stored and analyzed using the Statistical Package for the Social Sciences (SPSS) version 20.0. The normality of the variables was assessed using the Kolmogorov-Smirnov test. Median and interquartile ranges were used for variables that did not follow a normal distribution. The homogeneity of the sample regarding sociodemographic, clinical, habit, and disorder-related variables was assessed using Pearson's chi-square test.

The comparison of state anxiety mean scores between groups was performed using the Wilcoxon test. The comparison of the presence of anxiety precursors between groups was conducted using Pearson's chi-square test or Fisher's exact test, depending on the assumptions of each test. Intragroup comparisons of these signs and symptoms were conducted using the McNemar test.

All collected data are freely accessible for consultation at Mendeley Data^19^.

**Ethical considerations**

Approval for the study was obtained from the Ethics Committee of the University of International Integration of Afro-Brazilian Lusophony (approval number: 5.496.212). All national and international ethical guidelines for research involving human subjects were followed.

## Results

The study included 59 older adults, with 31 (52.54%) in the IG and 28 (47.45%) in the CG. Most participants in the IG were between 60 and 69 years of age, and between 60 and 74 years in the CG. Most participants were female, comprising 74.57% of the total sample (IG = 80.64%; CG = 67.85%), with a median income of R$1,212.00, and 50% of the respondents were retirees.

Regarding comorbidities, 16 (27.11%) participants had a diagnosis of diabetes mellitus (DM), and 34 (57.62%) had systemic arterial hypertension (SAH). The median duration of DM or SAH diagnosis among participants was 9 years, with a minimum of 1 year and a maximum of 15 years, as shown in Table 1.

The assessment of anxiety precursors, according to the associations made with the scales and the use of the intervention, shows that most symptoms before the intervention, except for three (cold hands, forgetfulness, and irritability), did not show significant differences between groups. After the intervention, significant differences were observed in three symptoms: impatience (p=0.014), fear (p=0.002), and anxiety (p=0.046), as shown in Table 2.

**Table 1:** Distribution of participants according to sociodemographic variables and pre-existing comorbidities. Redenção, Ceará, 2023. n=59

Sociodemographic variables and pre-existing comorbidities	IG (n=31) %(n)	CG (n=28) %(n)	p-value
Age range			0.474#
60 – 64 years old	32.25 (10)	32.14 (9)	
65 – 69 years old	29.03 (9)	25.00 (7)	
70 – 74 years old	16.12 (5)	28.6 (8)	
75 – 79 years old	6.45 (2)	7.14 (2)	
80 – 84 years old	9.70 (3)	3.57 (1)	
Over 85 years old	6.45 (2)	3.57 (1)	
Sex			0.816*
Male	19.35 (6)	32.14 (9)	
Female	80.64 (25)	67.85 (19)	
Occupation			0.827#
Retired	54.83 (17)	60.71 (17)	
Self-employed	12.90 (4)	14.28 (4)	
Other	32.25 (10)	25.00 (7)	
Marital status			0.579#
Married	45.16 (14)	57.14 (16)	
In a stable union	3.22 (1)	7.14 (2)	
Single	25.80 (8)	14.28 (4)	
Widowed	25.80 (8)	21.42 (6)	
Diabetes			0.728*
Yes	29.03 (9)	25.00 (7)	
No	70.96 (22)	75.00 (21)	
Systemic arterial hypertension			0.209#
Yes	61.29 (19)	53.57 (15)	
No	38.70 (12)	39.28 (11)	
Unknown	0 (0)	7.14 (2)	
Smoking			0.542#
Yes	16.12 (5)	10.71 (3)	
No	83.87 (26)	89.28 (25)	
Alcoholism			0.250*
Yes	16.12 (5)	28.57 (8)	
No	83.87 (26)	71.42 (20)	

IG: intervention group; CG: Control group. * Pearson's chi-square test; # Fisher's exact test.

In the intragroup comparison before and after the intervention, the CG did not show statistically significant differences. However, the IG exhibited significant differences in the following symptoms: impatience, fear, restlessness, sadness, and anxiety, all with p-values less than 0.001 (p<0.001), as shown in Table 3.

**Table 2:** Comparison of anxiety precursors before and after the intervention between groups. Redenção, Ceará, 2023.

Anxiety precursors	Before			After		
	Control Group %(n)	Intervention Group %(n)	p-value	Control Group %(n)	Intervention Group %(n)	p-value
Cold hands	7.14 (2)	29.03 (9)	0.031*	3.57 (1)	6.45 (2)	0.611#
Impatience	57.14 (16)	70.96 (22)	0.268*	53.57 (15)	22.58 (7)	0.014*
Fear	35.71 (10)	54.83 (17)	0.141*	32.14 (9)	3.22 (1)	**0.002#**
Tachycardia	39.58 (11)	48.38 (15)	0.482*	35.71 (10)	45.16 (14)	0.461*
Sweating	17.85 (5)	19.35 (6)	0.833*	17.85 (5)	12.9 (4)	0.597#
Anger	53.57 (15)	64.51 (20)	0.393*	43.42 (13)	32.25 (10)	0.265*
Tremors	10.71 (3)	25.80 (8)	0.137*	10.71 (3)	16.12 (5)	0.542#
Forgetfulness	60.71 (17)	83.87 (26)	0.046*	53.57 (15)	74.19 (23)	0.099*
Irritability	32.14 (9)	61.29 (19)	0.025*	32.14 (9)	25.80 (8)	0.592*
Helplessness	10.71 (3)	12.90 (4)	0.795#	7.14 (2)	3.22 (1)	**0.492#**
Restlessness	39.28 (11)	64.51 (20)	0.053*	32.14 (9)	19.35 (6)	0.260*
Tiredness	50.00 (14)	58.06 (18)	0.535*	42.85 (12)	45.16 (14)	0.859*
Sadness	35.71 (10)	54.83 (17)	0.141*	32.14 (9)	16.12 (5)	**0.201#**
Low Productivity	14.28 (4)	32.25 (10)	0.105*	10.71 (3)	22.58 (7)	**0.219#**
Anxiety	57.14 (16)	74.19 (23)	0.167*	39.28 (11)	16.12 (5)	0.046*
Loneliness	35.71 (10)	45.16 (14)	0.461*	28.57 (8)	12.90 (4)	0.135*

*Pearson's chi-square test; # Fisher's exact test.

**Table 3:** Comparison of intragroup anxiety precursors before and after the intervention. Redenção, Ceará, 2023.

Anxiety precursors	Control Group			Intervention Group		
	Before %(n)	After %(n)	p-value*	Before %(n)	After %(n)	p-value*
Cold hands	7.14 (2)	3.57 (1)	1.000	29.03 (9)	6.45 (2)	0.016
Impatience	57.14 (16)	53.57 (15)	1.000	70.96 (22)	22.58 (7)	**< 0.001**
Fear	35.71 (10)	32.14 (9)	1.000	54.83 (17)	3.22 (1)	**< 0.001**
Tachycardia	39.28 (11)	35.71 (10)	1.000	48.38 (15)	45.16 (14)	1.000
Sweating	17.85 (5)	17.85 (5)	1.000	19.35 (6)	12.90 (4)	0.500
Anger	53.57 (15)	46.42 (13)	0.500	64.51 (20)	32.25 (10)	0.002
Tremors	10.71 (3)	10.71 (3)	1.000	25.80 (8)	16.12 (5)	0.250
Forgetfulness	60.71 (17)	53.57 (15)	0.500	83.87 (26)	74.19 (23)	0.250
Irritability	32.14 (9)	32.14 (9)	1.000	61.29 (19)	25.80 (8)	**< 0.001**
Helplessness	10.71 (3)	7.14 (2)	1.000	12.90 (4)	3.22 (1)	0.250
Restlessness	39.28 (11)	32.14 (9)	0.500	64.51 (20)	19.35 (6)	**< 0.001**
Tiredness	50.00 (14)	42.85 (12)	0.500	58.06 (18)	45.16 (14)	0.125
Sadness	35.71 (10)	32.14 (9)	1.000	54.83 (17)	16.12 (5)	**< 0.001**
Low Productivity	14.28 (4)	10.71 (3)	1.000	32.25 (10)	22.58 (7)	0.250
Anxiety	57.14 (16)	39.28 (11)	**0.063**	74.19 (23)	16.12 (5)	**< 0.001**
Loneliness	35.71 (10)	28.57 (8)	0.500	45.16 (14)	12.90 (4)	0.002

*McNemar test.

The intragroup comparison of the STAI state anxiety subscale shows that the CG had little variation in mean and standard deviation before and after the intervention (p=0.398). In contrast, the IG showed a significant decrease in STAI state anxiety scores immediately after the intervention (p<0.001), as shown in Table 4.

**Table 4:** Comparison of intragroup STAI state anxiety mean scores before and after the intervention. Redenção, Ceará, 2023.

STAI	Before			After		
	CG	IG	p-value*	CG	IG	p-value*
State anxiety-M ± SD	39.82 ± 12.91	47.74 ±10.25	0.011	38.54 ± 12.46	34.32 ± 8.69	< 0.135

STAI: State-Trait Anxiety Inventory; CG (Control Group); IG (Intervention Group); M (Mean); SD (Standard Deviation) *Wilcoxon test.

**Figure 2:**
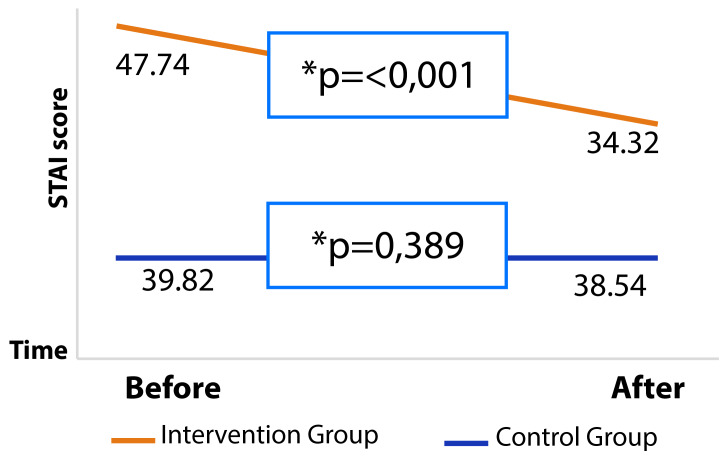
STAI state anxiety scores before and after intervention. Redenção, Ceará, 2023.

Based on the data obtained from the STAI instrument, it is possible to note that there was a significant improvement in the IG following the intervention, as can be seen in the curve in Figure 2. 

*Wilcoxon test.

## Discussion

The results reinforce that the use of flower therapy can produce beneficial effects on the anxiety levels of older adults, significantly reducing precursors such as impatience, fear, irritability, restlessness, sadness, and anxiety.

The benefits of integrative practices were also identified in another randomized controlled trial that followed 183 older adults from a rural community for 30 days, and divided participants into three groups (lavender, chamomile, and placebo). Aromatherapy with chamomile and lavender essential oils was found to improve anxiety, depression, and stress levels in older adults^20^.

The literature shows that the emergence of psychiatric disorders in older adults may vary, which can be due to sociodemographic factors in this population^21^,^22^. In this study, most of the older adults were female, between 60 and 69 years of age, married, non-white, retired, earning the minimum wage, and living with two or more family members.

Regarding the analysis of the clinical and epidemiological profile of older adults, it was found that aging, especially when related to illness, is influenced by lifestyle and by diagnoses of DM and SAH. Symptoms of depression and anxiety are present in older adults and can lead to the development of chronic non-communicable diseases (NCDs). A study conducted in Uganda showed that older adults with anxiety were 2.1 times more likely to develop an NCD^23^.

It was identified that 76.3% of the older adults did not have DM, which contradicts findings from several other studies on older adult populations^24^,^25^. Additionally, the prevalence of hypertension in the sample was higher than that of diabetes, 57% and 27% respectively, aligning with the global trend that predicts older adults are prone to hypertension^26^.

The results identified reinforce that flower therapy may yield beneficial effects on anxiety levels among older adults, with significant reductions in precursors such as impatience, fear, irritability, restlessness, sadness, and anxiety.

Educational strategies implemented within PHC to improve mental health in older adults are important tools for health promotion and for strengthening the bond between patients and the professional team^27^. Thus, these interventions emerge as enhancers of health care, considering the improvements in patients' physical and mental conditioning, as highlighted in this study, which showed reductions in fear and anxiety in the IG.

A study conducted in Spain with older adults observed a reduction in fear levels after implementing an intervention based on health education and exercise cycles. After one month of intervention, the IG showed decreased fear and improved balance^28^.

Regarding smoking and alcoholism, they were not prevalent in the intragroup sample, with percentages ranging from 83.8% to 89%. However, considering the correlation between anxiety and smoking, the study showed that older adult smokers exhibited higher anxiety levels compared to non-smokers, with a mean STAI state anxiety score of 39.63 for smokers and 35.80 for non-smokers.

In Ireland, a study conducted with older adults examined associations between smoking and smoking cessation in relation to generalized anxiety disorder (GAD) and found that smoking was associated with increased odds of GAD. Even individuals who had quit smoking demonstrated higher odds of GAD compared to those who had never smoked^29^.

Strategies that help reduce or quit smoking and other substance use can be implemented to improve anxiety symptoms. This was observed in a qualitative study involving 42 participants undergoing flower therapy, in which it was noted that flower essences reorganized emotional patterns, enhancing awareness of their habits and life changes, and enabling them to evaluate when something unusual was occurring and to take action using the therapy^30^.

Regarding physical activity, it was observed that physically active older adults had lower mean STAI state anxiety scores. Anxiety levels differed between sedentary and non-sedentary groups, indicating that physical activity promotes better mental health, enhances social relationships, and reduces risk factors for chronic diseases, strongly linking physical activity to decreased anxiety in older adults^31^.

This association was also noted in a study evaluating the mental and physical health of 166 older adult women in Australia who had no health problems preventing them from exercising. The results indicated that greater participation in physical exercise was associated with lower anxiety, and that anxiety sensitivity mediated this association^32^.

Using Neuman’s model to apply concepts and assumptions that facilitate interventions and care enables a holistic evaluation of the individual within the context of health and disease, helping identify stressors that contribute to anxiety^33^.

Agitation directly benefited from the intervention, as it was reduced in 85% of the older adults who reported experiencing it. However, sadness was one of the most challenging symptoms to compare and reduce, given that the STAI state anxiety subscale is answered based on how respondents feel at the moment of assessment and may therefore be influenced by recent experiences.

Additionally, anxiety levels in the IG decreased after the flower essence intervention (p-value: 0.135), confirming the positive relationship with the intervention. These findings align with the literature, which highlights the clinical applications of Bach flower therapy in reducing dental anxiety in children aged four to six years, improving behavior, and reducing pulse and blood pressure during dental procedures^34^.

Regarding the precursors of anxiety assessed during the nursing consultation, and in accordance with Betty Neuman's Systems Model and Bach's theory, the percentage of older adults reporting these precursors was much higher before the intervention, demonstrating a significant decrease in reported symptoms in the intragroup comparison after the intervention. It can be inferred that the reduction in the control group reflects what research suggests about therapeutic consultation or therapeutic rapport, which has been shown to reduce symptoms of depression and anxiety.

Interventions focused on the human essence, listening, and understanding implemented in a group reduce depressive and anxiolytic symptoms and improve mental health in older adults^35^.

The analysis of the use of flower therapy in reducing perceived stress levels among PHC nursing professionals demonstrated decreased stress at the end of the intervention^15^. Other reported benefits include increased awareness of self-perception and perception of surrounding events, improved interaction with the environment, greater emotional control, better mood, and reductions in anxiety and stress symptoms^36^,^37^. Additionally, a scoping review aimed at mapping the use of Bach flower essences in adult health care highlighted the use of four other essences in the included studies: Impatiens, Cherry Plum, White Chestnut, and Beech. Their effects, with the application of 4 diluted drops, contributed to reductions in anxiety, depression, fear, and pain^38^.

The use of complementary and integrative practices for anxiety reduction is part of various therapeutic approaches for patients with anxiety disorders. However, designing studies that demonstrate the effectiveness of each technique is crucial for the incorporation of protocols and practice gudelines^39^. Therefore, flower therapy should be used as a support tool within a holistic approach to anxiety reduction. When integrated into therapeutic protocols, flower essences offer a natural alternative that can be incorporated into anxiety treatment^9^.

The results of this study underscore the need for implementing non-invasive, effective, low-cost alternative practices without adverse effects for older adults, such as flower therapy^40^. Additionally, associating flower therapy with Neuman's Systems Model allows for a deeper analysis of anxiety predictors in older adults, as it identifies internal and external factors that may negatively affect the quality of life of the studied population^41^.

The limitations of this study were related to the non-use of flower remedies with patients in other healthcare services, as well as the fact that the study was conducted in only two health units within the same region, making it necessary to evaluate their use based on other variables.

## Conclusions

The use of flower essence therapy proved effective in reducing anxiety and its precursors, such as impatience, fear, irritability, restlessness, sadness, and anxiety, when comparing the IG and the CG.

It is therefore an innovative alternative for promoting older adults' health by improving their quality of life, encouraging health-promoting practices, and expanding the role of nursing in this context.

Incorporating this practice into primary health care can improve older adults' mental health, prevent chronic non-communicable diseases, and, consequently, achieve a better quality of life. It is relevant for future studies to expand the sample size and compare older adults from other regions, as well as to assess anxiety in other contexts using different types of flower essences.

## References

[1] Sousa  NF da S, Lima  MG, Cesar  CLG, Barros  MB de A (2018). Active aging: Prevalence and gender and age differences in a population-based study. Cadernos de Saúde Pública.

[2] Bezerra  PA, Nunes  JW, Moura  LBA (2021). Envelhecimento e isolamento social: uma revisão integrativa. Acta paul enferm.

[3] Huang  S, Wang  J, Zhang  Y, Qiu  Y, Wang  H, Yu  X (2024). Co-occurrence of depressive and anxious symptoms and their influence on self-rated health: a national representative survey among Chinese older adults. Aging & Mental Health.

[4] He  Z, Tan  WH, Ma  H, Shuai  Y, Shan  Z, Zhai  J (2024). Prevalence and factors associated with depression and anxiety among older adults: A large-scale cross-sectional study in China. Journal of affective disorders.

[5] Kitamura ES, Faria LR, Cavalcante RB, Leite ICG (2022). Depressão e transtorno de ansiedade generalizada em idosos pela infodemia de COVID-19. Acta Paulista De Enfermagem.

[6] Silva  L dos S, Sousa  AFD, Carvalho  DHF, Kalinke  LP (2023). Terapias não farmacológicas para pacientes oncológicos em Portugal e no Brasil: relato de experiência. Rev Esc Enferm USP.

[7] Møller  SR, Ekholm  O, Christensen  AI (2024). Trends in the use of complementary and alternative medicine between 1987 and 2021 in Denmark. BMC Complementary Medicine and Therapies.

[8] Marques  PP, Francisco  PMSB, Bacurau  AGM, Rodrigues  PS, Malta  DC, Barros NF (2020). Uso de práticas integrativas e complementares por idosos: pesquisa nacional de saúde 2013. Saúde em Debate.

[9] Ribeiro  J de A, Araújo  MHP de, Vieira  E da S, Maia  AED, Costa  DA da, Sousa  M do S (2020). Use of Floral Therapy in Anxiety and Stress. Braz. J. Hea. Rev. Curitiba.

[10] Silva  JPL, Morais  MST (2023). Flower Therapy in holistic care for the population during the COVID-19 pandemic. Saúde e Pesquisa.

[11] Zhong  Q, Niu  L, Chen  K, Lee  TM, Zhang  R (2024). Prevalence and risk of subthreshold anxiety developing into threshold anxiety disorder in the general population. Journal of Affective Disorders.

[12] Neuman  B, Fawcett  J (2011). The Neuman Systems Model.

[13] Penninx  BW, Pine  DS, Holmes  EA, Reif  A (2021). Anxiety disorders. Lancet.

[14] Fusco  SFB, Pancieri  AP, Amancio  SCP, Fusco  DR, Padovani  CR, Minicucci  MF (2021). Efficacy of Flower Therapy for Anxiety in Overweight or Obese Adults: A Randomized Placebo-Controlled Clinical Trial. J Altern Complement Med.

[15] Gava  FGS, Turrini  RNT (2024). Flower therapy and perceived stress in primary health care nursing professionals: randomized clinical trial. Rev Gaucha Enferm.

[16] Santos  KA da S, Cendoroglo  MS, Santos  FC (2017). Transtorno de ansiedade em idosos com dor crônica: frequência e associações. Revista Brasileira de Geriatria e Gerontologia.

[17] Roy-Byrne  PP, Davidson  KW, Kessler  RC, Asmundson  GJ, Goodwin  RD, Kubzansky  L (2008). Anxiety disorders and comorbid medical illness. General hospital psychiatry.

[18] Salles  L Fortes, Silva  MJP (2012). Efeito das essências florais em indivíduos ansiosos. Acta Paul Enferm.

[19] Brito  OD (2025). “Terapias Florais”. Mendeley Data, V1.

[20] Spielberger  CD, Biaggio  A, Natalício  LF (1979). Inventário de ansiedade traço estado: manual de psicologia aplicada.

[21] Ebrahimi  H, Mardani  A, Basirinezhad  MH, Hamidzadeh  A, Eskandari  F (2022). The effects of Lavender and Chamomile essential oil inhalation aromatherapy on depression, anxiety and stress in older community-dwelling people: A randomized controlled trial. Explore.

[22] Mendes-Chiloff  CL, Lima  MCP, Torres  AR, Santos  JLR, Duarte  YO, Lebrão  ML (2018). Depressive symptoms among the elderly in são paulo city, brazil: Prevalence and associated factors (SABE study). Revista Brasileira de Epidemiologia.

[23] Oliveira  LM de, Abrantes  GG de, Ribeiro  G da S, Cunha  NM, Pontes  M de L de F, Vasconcelos  SC (2019). Solidão na senescência e sua relação com sintomas depressivos: revisão integrativa. Rev. Bras. Geriatr. Gerontol.

[24] Bulamba  RM, Nalugoda  F, Nkale  J, Kigozi  G, Ochieng  AM, Kyasanku  E (2024). Examining associations between mental health and Chronic Non-Communicable Diseases (C-NCDs) among older adults in Wakiso, Uganda. PloS one.

[25] Matos  A, Calado  M, Mendes  M, Pedrosa  S, Figueiredo  M do C (2020). Educação para a saúde aos idosos com diabetes mellitus: uma scoping Review. Revista da UIIPS- Unidade de Investigação do Instituto Politécnico de Santarém.

[26] Melo  EG, Santos  CLJ, Santos  J, Batista-Filho  RA, Souza LL Vasconcelos DS, Lima  ACC (2019). Perfil sociodemográfico e clínico de idosos com diabetes. Rev enferm UFPE.

[27] Bezerra  HC de J, Gaudêncio  E de O, Batista  JR de M, Lucena  M do SR, Oliveira  AR (2021). A relação hipertensão arterial, ansiedade e estresse: uma revisão integrativa de literatura. Psicol. estud.

[28] Souza  AP de, Rezende  KTA, Marin  MJS, Tonhom  SF da R, Damaceno  DG (2022). Ações de promoção e proteção à saúde mental do idoso na atenção primária à saúde: uma revisão integrativa. Ciência & Saúde Coletiva.

[29] Meléndez-Moral  JC, Garzón-Soler  T, Sales-Galán  A, Mayordomo-Rodríguez  T (2014). Efectividad de una intervención para reducir el miedo a caer en las personas mayores. Aquichan.

[30] Monroe  DC, McDowell  CP, Kenny  RA, Herring  MP (2021). Dynamic associations between anxiety, depression, and tobacco use in older adults: Results from The Irish Longitudinal Study on Ageing. Journal of psychiatric research.

[31] Pancieri  AP, Fusco  SB, Ramos  BIA, Braga  EM (2018). Significados da terapia floral para ansiedade em pessoas com sobrepeso ou obesidade. Rev Bras Enferm.

[32] Pawlina  MMC, Rondina  R de C, Espinosa  MM, Botelho  C (2015). Depressão, ansiedade, estresse e motivação em fumantes durante o tratamento para a cessação do tabagismo. J. Bras. Pneumol.

[33] Nah  K, Olthuis  JV (2023). Exercise and Health Anxiety in Older Women: Exploring the Mediating Role of Anxiety Sensitivity. Journal of Aging and Physical Activity.

[34] Lima  LH de SS, Monteiro  EMLM, Coriolano  MW de L, Linhares  FMP, Cavalcanti  AMT de S (2020). Fortalezas familiares na Síndrome Congênita do Zika à luz de Betty Neuman. Revista Brasileira de Enfermagem.

[35] Dixit  UB, Jasani  RR (2020). Comparação da eficácia da terapia floral de Bach e da musicoterapia na ansiedade odontológica em pacientes pediátricos: Um estudo controlado. Revista da Sociedade Indiana de Odontopediatria e Odontologia Preventiva.

[36] Souza  AP de, Rezende  KTA, Marin  MJS, Tonhom  SF da R, Damaceno DG (2022). Mental health promotion and protection actions aimed at the elderly in the context of primary health care: an integrative review. Ciência & Saúde Coletiva.

[37] Rivas-Suárez  SR, Águila-Vázquez  J, Suárez-Rodríguez  B, Vázquez-León  L, Casanova-Giral  M, Morales-Morales  R (2015). Exploring the effectiveness of external use of bach flower remedies on carpal tunnel syndrome: a pilot study. J Evid Based Complementary Altern Med.

[38] Siegler  M, Frange  C, Andersen  ML, Tufik  S, Hachul  H (2017). Effects of bach flower remedies on menopausal symptoms and sleep pattern: a case report. Altern Ther Health Med.

[39] Rocha  MA, Queiroz  CG, Dos Santos  KVG, dos Santos Dantas  JK, Araujo  SCM, Dutra  SVO (2022). Bach Flower Remedies as Complementary Therapies in Health Care: A Scope Review. Holist Nurs Pract.

[40] NG  JY, Parakh  ND (2021). A systematic review and quality assessment of complementary and alternative medicine recommendations in insomnia clinical practice guidelines. BMC complementary medicine and therapies.

[41] Pitilin  EB, Sbardelotto  T, Soares  RB,  Resende  TC, Tavares  D, Haag  F (2022). Terapia floral na evolução do parto e na tríade dor-ansiedade-estresse: estudo quase-experimental. Acta Paulista de Enfermagem.

